# Mesenteric adipose tissue: from protective gatekeeper to driver of inflammatory bowel disease

**DOI:** 10.1097/in9.0000000000000081

**Published:** 2026-05-14

**Authors:** Christy M. Gliniak

**Affiliations:** 1 Department of Nutritional Sciences, Rutgers, the State University of New Jersey, New Brunswick, NJ, USA

**Keywords:** mesenteric adipose tissue, inflammatory bowel disease, lymph nodes, immune cells, adipocytes, adipocyte progenitors

## Abstract

Inflammatory bowel disease (IBD) defines a group of diseases, including Crohn’s disease and ulcerative colitis, characterized by chronic inflammation of the gastrointestinal tract. Evidence suggests that visceral adipose tissue, particularly its mesenteric component, influences the course of IBD through its immunomodulatory properties. Mesenteric adipose tissue (MAT) is composed of multiple cell types, including adipocytes, preadipocytes, and immune cells, that collectively regulate energy balance and endocrine signaling. MAT is a unique fat depot as it directly connects along most of the intestinal serosa and harbors the arteries, veins, and lymph nodes that support intestinal function. MAT’s placement at the intestinal barrier serves to contain microbial translocation and support the repair of intestinal epithelium. However, inflammation initiated by obesity or IBD renders it maladaptive, triggering an influx of immune cells that, in turn, release proinflammatory adipokines, cytokines, and chemokines, resulting in local and systemic metabolic effects. Clinical data strongly link the accumulation of inflammatory MAT in the pathology of Crohn’s disease through the development of “creeping fat", a fibrotic, immune-rich tissue that shields the inflamed bowel but can also exacerbate the condition. The inflammatory mediators produced by MAT and the mechanisms by which they aggravate IBD are not well understood. This review synthesizes recent advances in the understanding of the cellular complexity of MAT, emphasizing the bidirectional immune crosstalk between the intestine and the mesentery, and exploring how metabolic or microbial stress drives maladaptive transformation. Finally, we highlight therapeutic strategies that may preserve the reparative functions of MAT while limiting fibrogenic remodeling.

## 1. Introduction: mesenteric adipose tissue at the gut–systemic interface

Over the past two decades, there has been a dramatic increase in the prevalence of inflammatory bowel disease (IBD), coincident with the obesity epidemic ^[1,2]^. IBD is expected to affect 1 in 100 individuals in certain populations in the near future, especially in Western countries and newly industrialized countries, which are also showing similar developments ^[3,4]^. An overlap of metabolic diseases and IBD is expected to be observed in the future. Mesenteric adipose tissue (MAT) occupies an exclusive anatomical and immunological position that distinguishes it from all other fat depots. As a visceral fat depo embedded within the mesentery, its abdominal proximity to the gut, lymphatic drainage, and portal circulation places MAT at the center of a highly integrated metabolic–immune network ^[5]^. Unlike other visceral fat pads, which primarily function in energy storage and endocrine signaling, MAT directly encounters dietary metabolites, microbial products, and signals released from stressed or damaged intestinal epithelium ^[6]^. This proximity positions MAT to be protective, containing microbial translocation, but it also creates vulnerability: under chronic stimulation, the same pathways become dysregulated, driving fibrosis, immune activation, and systemic metabolic disturbance.

### 1.1 Mesenteric adipose tissue as a specialized immune organ

**Figure 1.**
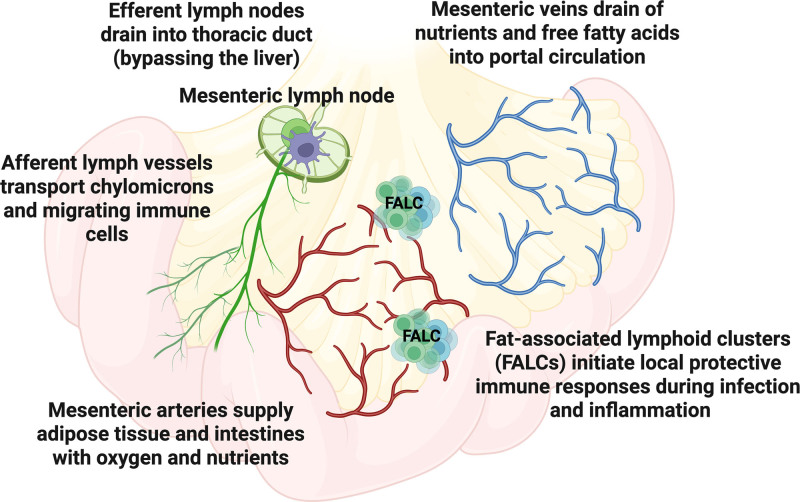
**Structural and vascular organization of MAT**. MAT lies between the gut and liver and directly connects the intestinal serosa via a continuous interface along most of the small and large bowels. MAT harbors the mesenteric arteries, veins, lymph vessels and nodes, and nerves that support intestinal function. Mesenteric arteries supply adipose tissue and the intestines with oxygen and nutrients, while mesenteric veins drain nutrients, metabolites, and free fatty acids into the portal circulation. Afferent lymphatic vessels transport antigens, immune cells, and chylomicrons from the intestine to MLN, which filter lymph, removing intestinal pathogens, dietary antigens, and cellular debris. MLN are primary sites for T and B cell activation to create immune tolerance to food and defend against harmful microbes, and drain via efferent lymphatics into the thoracic duct, bypassing the liver. FALCs are distributed throughout the mesenteric adipose tissue and initiate local protective immune responses and antibody production during infection and inflammation. Figure created using biorender.com. FALC, Fat-associated lymphoid clusters; MAT, mesenteric adipose tissue; MLN, mesenteric lymph nodes.

The mesentery is a fold of the peritoneum that connects the intestine to the posterior abdominal wall ^[5]^. This continuous organ contains the blood vessels, lymphatics, nerves, and adipose tissue that support the function of the gastrointestinal tract (Figure 1). Although the mesentery was identified centuries ago, with one of the earliest depictions made by Leonardo da Vinci circa 1508, it had long been described in medical texts as a fragmented structure composed of disconnected parts ^[7]^. In 2012, J. Calvin Coffey and his team published a landmark study demonstrating that the mesentery is a continuous organ with a clearly defined structure ^[5,8]^. This reclassification marked a significant advancement in understanding gastrointestinal physiology and sparked growing interest in investigating the mesentery beyond its traditional role as a connective tissue.

### 1.2 Proximity to the gut shapes MAT function

MAT directly attaches to the intestinal serosa, placing it in close proximity to the gut microbiota and their metabolites ^[9]^. This positioning allows MAT to serve as a defensive barrier against the trillions of microorganisms residing in the intestine that can cross the intestinal epithelial barrier ^[10]^. Furthermore, MAT exhibits a distinctive immune cell profile compared with other adipose tissue depots, mediating local and systemic immune responses ^[11–13]^. It is becoming clear that the intestinal and MAT immune systems cooperate to continually sustain a balance between immunity to pathogens and tolerance of commensal bacteria and other factors.

### 1.3 MAT is a tissue with a role in protection and pathology

MAT anatomy is also unique in that it drains directly into the portal vein, exposing the liver to high concentrations of substances released by this fat depot ^[14]^. This anatomical relationship provides one explanation for why visceral adiposity is strongly associated with liver dysfunction and systemic metabolic diseases such as type 2 diabetes ^[6]^. Indeed, numerous studies have demonstrated that expansion of abdominal visceral adipose tissue during obesity is associated with the incidence and progression of inflammatory diseases ^[15–17]^. During chronic overnutrition, adipocytes expand to the point that they become hypoxic and secrete inflammatory factors ^[18]^. Moreover, immune cells residing within adipose tissue, which constitute the second-largest cellular component after adipocytes, undergo significant changes in number and activity during obesity, thereby activating local and systemic inflammatory responses ^[19]^. Under pathological conditions such as obesity and IBD, MAT’s protective capabilities diminish, transforming it into a driver of chronic inflammation that contributes to systemic complications, including insulin resistance and cardiovascular disease ^[6,20,21]^, and also exacerbates autoimmune diseases, inflammatory conditions like IBD and asthma, chronic fatigue, or liver disease ^[22]^.

In this review, we describe the distinct anatomy and cellular composition of MAT and summarize the current literature on how it confers its protective or destructive role in metabolic immunity. We discuss the evidence for whether MAT drives chronic inflammation within the context of obesity and IBD. Given the limited therapeutic options for IBD, we highlight literature suggesting that targeting mesenteric fat could offer new strategies for mitigating inflammation-related disorders.

## 2. Structural and functional organization of mesenteric adipose tissue

MAT shares core features with other fat depots, such as lipid storage and endocrine activity. Its anatomical attachment to the intestinal serosa and contact with mesenteric lymph nodes (MLNs) place it at the interface of gut, immune, and metabolic signaling ^[23–25]^. As a result, MAT continuously integrates inputs from the intestinal epithelium, microbial metabolites, neural pathways, and circulating nutrients ^[14,26–28]^. These features position MAT as both a metabolic organ and an immunological hub, capable of responding rapidly to changes in gut physiology, barrier integrity, and systemic metabolic state. Importantly, the mesentery and its associated adipose tissue are anatomically comparable between mice and humans, making rodent models informative for understanding MAT biology in the context of human disease. Both species possess a continuous mesenteric structure that supports the intestinal vasculature and lymphatics, and harbor similar cell populations within the tissue ^[5,29]^. The following sections outline the unique vascular, lymphatic, and regional characteristics of MAT that collectively define its specialized roles in gastrointestinal and systemic homeostasis.

### 2.1 MAT vasculature

The intestine requires a substantial and adaptable blood supply, delivered by the superior and inferior mesenteric arteries and veins. As a result, MAT is one of the most highly vascularized visceral fat depots in both mice and humans ^[18]^. This extensive vascularization of MAT facilitates the delivery of oxygen and nutrients, enabling continuous signaling between endothelial cells and surrounding stromal and immune cells. Crosstalk between MAT and its vasculature helps maintain vascular tone, supporting digestion, nutrient absorption, and epithelial barrier integrity ^[30,31]^.

Endothelial dysfunction in MAT is both a consequence and a contributor during metabolic and inflammatory stress ^[18]^. Under these conditions, endothelial cells adopt a proinflammatory phenotype, producing cytokines such as tumor necrosis factor-alpha (TNF-α) and interleukin-6 (IL-6), which amplify local inflammation and increase levels of circulating inflammatory mediators. Disruption of vascular function in MAT can also increase intestinal permeability, facilitating the infiltration of immune cells or their cytokines into adipose tissue.

Anatomically, MAT lies directly between the intestine and the liver. The superior and inferior mesenteric veins drain into the portal vein ^[32]^. This means that MAT-derived cytokines, metabolites, and absorbed nutrients initially reach the liver, influencing hepatic inflammation, glucose and lipid metabolism, and systemic immune tone ^[33]^. Healthy mesenteric fat can buffer microbial and metabolic signals before they reach the liver, whereas dysfunctional MAT may worsen intestinal inflammation and contribute to hepatic and systemic metabolic dysregulation.

It is worth noting that mesenteric perivascular adipose tissue (PVAT), the fat immediately surrounding mesenteric blood vessels, represents a specialized and often continuous compartment within MAT that directly interfaces with the vasculature. Mesenteric PVAT harbors distinct immune cell populations, including T cells and macrophages, that undergo phenotypic shifts during metabolic stress and can influence vascular tone and function ^[34]^. In experimental colitis, mesenteric PVAT undergoes adipose remodeling and leukocyte accumulation, which promote mesenteric vascular dysfunction ^[35]^. Although a comprehensive discussion of PVAT is beyond the scope of this review, these findings underscore that the immunological and vascular functions of mesenteric fat extend to the perivascular compartment and warrant further investigation in the context of IBD.

Given this unique vascular and anatomical positioning, understanding the bidirectional communication between the mesenteric vasculature and the diverse cell types in MAT is critical for developing targeted therapeutic strategies for chronic inflammatory conditions, such as obesity and IBD.

### 2.2 Lymphatic networks in mesenteric fat

The lymphatic system comprises highly organized structures distributed throughout the lymphatic vessel network at strategic sites in the body ^[36]^. Intestinal lymphatic vessels collect lymph fluid, including absorbed dietary lipids, and transport it back to the bloodstream. Within MAT, these lymph vessels connect to an extensive network of lymph nodes that collect pathogens that escape local elimination, thereby establishing a second line of defense against bacteria in the gut ^[16]^. They moderate immunity by collecting antigens and antigen-presenting cells from surrounding tissues, thereby providing an effective environment for antigen presentation to lymphocytes and for the generation of memory immune responses. Intriguingly, most lymph nodes are embedded in adipose tissue, although the reasons for this remain unclear ^[37]^. The MLNs are the most studied aspect of MAT immunity, no doubt due to their association with obesity and IBD. Notably, MLNs are immunologically distinct from peripheral lymph nodes, as cells such as dendritic cells display different homing markers for each type of lymph node ^[38,39]^. This anatomical compartmentalization of lymphatic drainage is important for intestinal homeostasis, allowing for appropriate priming of tolerogenic or effector immune responses, such as the ability to tolerate oral antigen while eliciting protective effector responses toward pathogens ^[40]^.

While lymphatic vessels are designed to transport this fluid efficiently, there is a level of exposure to surrounding cells and tissues, including the mesenteric fat and immune cells, to components within the lymph (lipids, antigens, and immune cells). Thus, MLNs and MAT experience steep, pulsatile gradients of lipoproteins and their contents, as well as microbial products such as lipopolysaccharide (LPS) metabolites, bile acids, and cytokines, which are unlike those found in any other tissue of the body. This exposure is a normal part of the body’s immune surveillance process, but it can become dysregulated during disease. For example, postprandial dietary lipids and chylomicron remnants may carry LPS into mesenteric lymphatics ^[41]^. And a fraction of chylomicron fatty acids escapes from the lymphatics into the systemic nonesterified fatty acid pool ^[42]^.

MAT contains nonclassical lymphoid clusters of hematopoietic cells, fat-associated lymphoid clusters (FALCs), which surround adipocytes ^[43]^. FALCs are also known as milky spots, as they are visual aggregates of white blood cells, particularly innate B-1 cells and macrophages ^[43]^. FALCs are found in both the human greater omentum and the mouse abdominal fat ^[44]^. FALCs are not encapsulated like a lymph node; thus, they are in direct contact with surrounding adipocytes ^[45]^. They increase in number and size in response to a high-fat diet or peritoneal inflammation ^[45]^. The cellular composition of FALCs in healthy and inflamed MAT is discussed in *Section 3.3*. Despite their likely importance in coordinating local immune responses, the functional significance of the intimate association between FALCs and mesenteric adipocytes remains poorly understood.

It is important to distinguish FALCs from crown-like structures (CLS), which are histologically defined aggregates of macrophages that surround dead or dying adipocytes during obesity-driven adipose tissue dysfunction ^[46]^. CLS reflect an injury response, as macrophages are recruited to clear necrotic adipocytes, a hallmark of chronic low-grade inflammation and fibrosis in metabolically unhealthy fat depots ^[46]^. In contrast, FALCs are organized lymphoid aggregates enriched in innate B-1 cells and group 2 innate lymphoid cells (ILC2s) that serve an immune surveillance function, producing natural antibodies and coordinating mucosal defense ^[47]^. While CLS expands in proportion to adipocyte death and tissue damage, FALCs have been shown in mice and humans to increase in number and size in response to infection or peritoneal inflammation and are constitutively present in healthy mesenteric fat ^[44,48]^.

### 2.3 Regional specialization along the gastrointestinal tract

The characteristics of MAT vary significantly across different segments of the gastrointestinal tract, reflecting the distinct physiological functions and immunological environments of these regions ^[49]^. The small and large intestines have distinct structures, tissue composition, and microbiota ^[50]^. The large intestine lacks villi and is optimized for the absorption of water, dissolved electrolytes, and metabolites produced by the microbiota. The small intestine is responsible for nutrient absorption and B-cell-mediated food tolerance ^[51]^. This distinction is also reflected in MAT, which exhibits distinct characteristics along different intestinal segments and varied roles in immune regulation ^[49]^. For example, the MLNs draining the small intestine and colon are anatomically separate and immunologically distinct ^[39]^. Most studies of mesenteric fat do not distinguish between regions associated with the small vs the large intestine, making their findings difficult to interpret in an immunological context.

## 3. Cellular heterogeneity defines MAT physiology

We are just beginning to understand the factors that govern the complex cellular networks in MAT and how they change in response to stressors like obesity and IBD. Generally, the expansion of white adipose tissue is regulated by coordinated interactions between adipocytes and the stromal vascular fraction, which comprises endothelial cells, nerve fibers, immune cells, adipocyte progenitors, and other cell types. In MAT, adipocytes form the bulk of the tissue, followed by the hematopoietic compartment, which comprises multiple lymphoid subsets, including T cells, B cells, plasma cells, invariant natural killer T cells, and ILC2s. Recent advances in single-cell and spatial transcriptomic technologies have uncovered striking depot-specific heterogeneity, revealing that cells within the MAT possess distinct properties compared with those in other visceral fat depots ^[12,52–54]^. These findings have set the stage for a new understanding of how immune and stromal plasticity within MAT governs its transition between protective and pathogenic states, influencing both intestinal and systemic disease outcomes.

### 3.1 Adipocyte heterogeneity in MAT

Adipose tissue distribution in the human body is highly heterogeneous, and there are also adipocyte subtypes that exhibit functional specialization. Current single-cell RNA sequencing workflows struggle to capture large cells, such as adipocytes, so insights into adipocyte subtypes are primarily derived from single-nuclei datasets. Using this approach, Xie et al ^[13]^ characterized gene expression and physiological properties of adipocytes across five depots in mice under normal physiological conditions, revealing significant heterogeneity and multiple cellular lineages of origin ^[13]^. Within MAT, adipocytes display functional and morphological diversity influenced by their anatomical location and proximity to immune cells. Notably, WNT type-1 adipocytes are preferentially concentrated in MAT adjacent to the ileum and colon, regions prone to inflammation ^[13]^. The colon harbors a dense microbiome, relies on a dual-layered mucus barrier, and lacks Peyer’s patches; together with the terminal ileum, it represents a common site for IBD. The enrichment of specific adipocyte populations in these regions suggests that they may influence local immune properties. However, the precise mechanisms by which these adipocytes exert immunomodulatory effects, particularly in the context of obesity, altered intestinal permeability, and microbial translocation, require further investigation.

### 3.2 Heterogeneity of stromal vascular cells in MAT

The adipose stromal vascular fraction is a diverse cell population comprised of endothelial cells, pericytes, smooth muscle cells, and various types of stem cells. Collectively, these cells can spontaneously form vessel-like networks in vitro and robust, functional vasculatures in vivo. Pericytes are mesenchymal-derived mural cells, important for blood vessel development, function, and stability. In comparison to other white adipose tissues, pericytes were significantly increased in MAT, whereas adipocytes were drastically diminished ^[13]^. They interact with endothelial cells to stabilize blood vessels and support vascular development and maintenance. Endothelial cells further promote vascular development by releasing signaling molecules, including growth factors and extracellular matrix proteins, thereby facilitating the growth and maturation of new blood vessels. Notably, the MAT depot possesses a greater proportion of lymphatic endothelial cells compared with other fat pads, a feature potentially relevant to the increased lymphatic vascular growth and dysfunction observed in MAT ^[13]^. In the MAT, highly specialized endothelial cells are adapted to regulate lipid transport and secrete lipids that activate peroxisome proliferator–activated receptor (PPAR) gamma, thereby regulating adipose cell function ^[55]^.

The adherent stromal vascular fraction of adipose tissue also contains highly proliferative and multipotent adipose-derived stem cells ^[56]^. These stem cells, often found within blood vessels themselves, serve as a reservoir for mesenchymal stem cells capable of differentiating into adipocyte progenitor cells and other cell types ^[57]^. Lineage-committed “preadipocytes” are destined for terminal differentiation into mature adipocytes. The same adipocyte progenitor subtypes are present in visceral fat depots, including MAT ^[13,57,58]^. Compared with other depots, adipose stem cells from subcutaneous white adipose tissue and MAT demonstrated the strongest adipogenic and myogenic differentiation capabilities, respectively ^[13]^.

Intriguingly, unlike other fat pads, increased PPAR gamma activity in MAT preadipocytes may not induce adipogenesis, suggesting that the transcriptional regulation of adipogenesis in MAT is distinct from other visceral adipose depots ^[57]^. Furthermore, stromal and immune cell crosstalk in MAT plays a critical role in intestinal immunity, particularly in integrating signals from bacteria, their metabolites, or damaged or infected epithelial cells ^[59]^. Batra et al ^[60]^ determined that mouse mesenteric preadipocytes were competent in phagocytosing a broad range of antigens, acting as a defense mechanism against bacteria that translocate into the mesenteric fat. However, unlike macrophages, mesenteric preadipocytes do not express major histocompatibility complex class II (MHC II) on their surface, indicating that preadipocytes do not function as antigen-presenting cells in the fat tissue ^[60]^.

### 3.3 Immune cell diversity in MAT

Immune cells within MAT reside among the adipocytes, and because they are located near the intestinal epithelial layer, they act as a first line of defense against pathogens that cross the intestinal barrier. MAT contributes to immune surveillance by filtering antigens and microbial products that enter the mesenteric lymphatics, supporting the generation of protective immune responses while promoting tolerance to food antigens and commensal bacteria. Table 1 provides an overview of the primary immune cell types identified within MAT lymphoid structures, outlining their proposed functions ^[66–68]^. The subsequent sections explore MAT’s unique depot-specific immune landscape and the critical functional, adaptive, and maladaptive effects within MAT, highlighting their profound impact on intestinal and systemic health.

**Table 1:** Primary immune cell types identified within mesenteric adipose tissue lymphoid structures.

	Mesenteric lymph nodes	Mesenteric adipose tissue/fat-associated lymphoid clusters
Description	These are encapsulated, highly organized secondary lymphoid organs that act as central hubs for immune surveillance, filtering lymph from the gastrointestinal tract and mesentery. They have distinct B-cell and T-cell zones and are connected by both afferent and efferent lymphatic vessels. Krishnamurty & Turley 2020 ^[38]^.	Immune cells in MAT reside within the adipose tissue itself, often in nonencapsulated clusters called FALCs. These clusters are in direct contact with surrounding adipocytes and are highly vascularized but have less defined lymphatic connections compared with conventional lymph nodes. Jackson-Jones et al 2016 ^[61]^.
Overall composition	High density of immune cells, primarily lymphocytes, organized into distinct regions (cortex, paracortex, medulla). Macpherson and Smith 2006 ^[62]^.	Lower overall immune cell density relative to the mass of the tissue; rich in tissue-resident cells. Benezech et al 2015 ^[45]^.
Major lymphocyte types	T cells and B cells are both abundant, with T cells and dendritic cells mainly in the paracortex and B cells in the follicles. Esterhazy et al 2019 ^[40]^.	B cells are often the majority of lymphocytes present, including B1 B cells. Srikakulapu & McNamara 2020 ^[63]^; Benezechet al 2015 ^[45]^.
B Cells	Activated B cells migrate from the MLN back to the intestinal lamina propria to provide localized immune protection. Dubey et al 2017 ^[64]^.	B1 cells, which produce natural antibodies (IgM) without prior activation, providing rapid protection. They also support adaptive immune responses via B2 cells. Daley & Benezech 2024 ^[47]^.
T cells	Regulatory T cells (Tregs). Contains a high proportion of conventional T helper (CD4^+^) cells and cytotoxic T (CD8^+^) cells. Subpopulations of CD8^+^ T cells and CD3^+^CD11c^+^ innate lymphocytes are present in MLN but not MAT. Cording et al 2013 ^[65]^.	Contains unique populations of visceral adipose tissue-associated regulatory T cells. Appears to be no defined T-cell zone. Moro et al 2010 ^[48]^; Cruz-Migoni & Caamano et al 2016 ^[44]^.
NKT cells	Lower frequencies but NKT cells may play roles in modulating classical T-cell responses. Lee et al 2015 ^[66]^.	Higher frequencies of CD1d-restricted NKT cells. Benezech, et al 2015 ^[45]^.
Innate lymphoid cells	ILC1-3 present, and ILC3 dominate. Kastele et al 2021 ^[67]^.	Notably contains type 2 ILCs Jackson-Jones et al 2016 ^[61]^.
Macrophages & dendritic cells	Abundant antigen-presenting cells. Gross, Salame, and Jung 2015 ^[68]^.	Abundant macrophages and dendritic cells lack follicular dendritic cells. Daley & Benezech 2024 ^[47]^.

FALC, Fat-associated lymphoid clusters; ILC, innate lymphoid cells; MAT, mesenteric adipose tissue; MLN, mesenteric lymph nodes; NKT, natural killer T.

#### 3.3.1 Innate immunity in mesenteric fat

Innate immunity is the rapid, nonspecific, first-line defense system that detects and quickly responds to “environmental signals” in the tissue. In adipose tissue, innate immune cells, such as macrophages, natural killer cells (NK), and ILC2s, engage to maintain tissue homeostasis ^[69,70]^. The recruitment, proliferation, activation, and polarization of innate immune cells in visceral fat depots, such as gonadal white adipose tissue located around the reproductive organs, follow an overlapping but recognizable timeline during the development of obesity. However, the sequence of these events has not been systematically characterized in MAT, partly because few studies have examined this depot under healthy conditions. Nevertheless, the available literature does reveal depot-specific differences in innate immune cell composition across species, including rodents and cattle.

Within MAT, macrophages and other immune cells reside adjacent to adipocytes or within FALCs. Macrophages engulf and clear pathogens, cellular debris, and bacterial products, thereby providing local and systemic protection against pathogenic bacteria while maintaining tolerance of commensal bacteria. In mice on a chow diet, macrophages in MAT were predominantly M2-like macrophages ^[13]^. It has been demonstrated in mice that MAT contains more resident macrophages and fewer lipid-associated macrophages compared with visceral epididymal white adipose tissue, whereas T cells and dendritic cells show minimal differences among depots ^[13]^. Macrophage biology in healthy MAT has been more extensively characterized in cattle, where macrophages and dendritic cells exhibit similar properties whether sampled from MAT or MLNs ^[29,30]^.

Emerging evidence suggests that MAT immunity is dynamically regulated by nutrient intake. MAT macrophages appear sensitive to dietary fatty acids: oleic acid supplementation increases M2 macrophages in murine MAT ^[71]^, and *n*-3 fatty acids exert distinct metabolic and immunomodulatory effects in mesenteric vs epididymal adipose tissue ^[71,72]^. These observations suggest that MAT immune responses are coordinated with feeding to regulate the influx of dietary lipids and microbial products that accompany nutrient absorption. However, the mechanisms by which this balance is achieved remain poorly understood.

FALCs are enriched in innate B cells and ILC2 cells that secrete natural antibodies, playing a crucial role in the early control of infection and serving as a defense against the gut microbiota ^[45,63]^. ILCs are the innate counterparts of T cells, but they lack antigen-specific receptors. The first description of ILC2 as a unique cell population was as resident cells of the mesenteric fat ^[48,73]^. Over the last decade, ILCs have emerged as major regulators of type 2 immunity in adipose tissue, coordinating the function of eosinophils, macrophages, adipocytes, and innate type B cells ^[69]^. Distinct ILC subsets activate different immune responses, and their importance has been observed across adipose tissue depots. ILC2s are reported in most fat depots ^[74]^ and are now considered key regulators of human adipose tissue homeostasis ^[75]^, although less is known specifically about their function in MAT.

The innate-like B-cell populations (B1 cells) in FALCs from mesenteric and visceral fat that produce natural antibodies essential for early infection control and protection against autoimmunity. Natural antibodies produced by B1 act as “eat-me” signals for the phagocytosis of cellular debris and invading pathogens. In chow-fed mice, the MAT depot contained the most abundant B-cells vs epididymal or subcutaneous adipose tissue ^[13]^. FALC stromal cells secrete chemokines that recruit and retain B cells in clusters where ILC2s are also present, coordinating their interactions and enabling localized mucosal protection by producing innate antibodies ^[45]^. How these immune cell populations integrate their responses within MAT remains an important open question.

#### 3.3.2 Adaptive immunity in MAT

The adaptive immune system, comprising T and B lymphocytes, has historically received less attention in the context of MAT immunobiology. Unlike innate immunity, the adaptive immune system provides antigen-specific responses, long-term immunological memory, and refined control over inflammatory processes. Within the gut-draining MLNs, a unique microenvironment promotes site-specific immune responses distinct from those generated in peripheral lymph nodes ^[40,62,76]^. T cells primed in the MLN are imprinted to express gut-homing receptors, including α4β7-integrin and the chemokine receptor CCR9, enabling their specific migration to the small intestine ^[77]^. It is speculated that, due to their unique niche, MAT immune cells may be primed and distinct from other fat depots.

T lymphocytes are key components of adaptive immunity within MAT. In healthy adipose tissue, regulatory T-cells and T helper type 2 cells are abundant and essential for maintaining immune tolerance, the active suppression of inflammatory responses to self-antigens and harmless environmental antigens, including those derived from commensal bacteria and dietary components ^[49]^. This tolerogenic environment is critical for preventing inappropriate immune activation in a tissue continuously exposed to gut-derived signals ^[65]^. Single-cell and flow cytometry analyses in mice fed a standard chow diet revealed that T-cell populations exhibit minimal differences among adipose depots ^[13]^. However, in Holstein-Friesian cows, the frequency of both γδ T-cells and CD8^+^ T non-γδ T-cells is higher in mesenteric than in subcutaneous adipose tissue, suggesting species-specific or depot-specific variations in T-cell distribution ^[78]^.

B cells mediate humoral immunity through antibody production, and distinct subsets exert opposing effects on inflammation. Regulatory B cells produce anti-inflammatory cytokines, whereas other subsets can produce proinflammatory mediators such as interferon-gamma (IFN-γ) during obesity. In lean mice, MAT harbors the most abundant B cell population among fat depots, although classical B2 cells are notably absent ^[13]^. B cells can migrate from MAT to the liver, a process that becomes more pronounced during high-fat diet feeding ^[79]^. Emerging data indicate that B lymphocytes infiltrate MAT early in the development of nonalcoholic fatty liver disease (NAFLD), where they may promote local inflammation by regulating macrophages and subsequently migrate to the liver to induce hepatocyte inflammation ^[79]^. Conversely, evidence suggests that MAT-derived B cells may also migrate to the intestine and exacerbate colitis in rodent models ^[80]^. Human data confirming these trafficking patterns remains limited.

## 4. Development of maladaptive mesenteric fat

**Figure 2.**
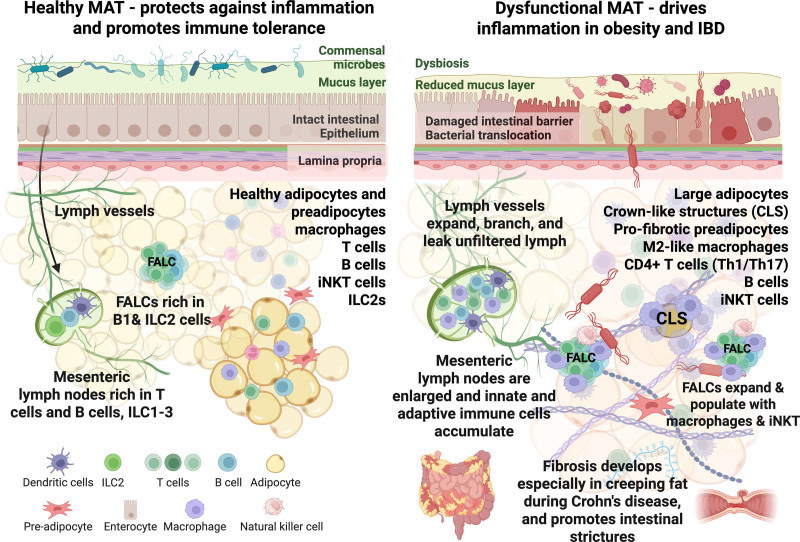
**Healthy vs dysfunctional mesenteric adipose tissue.** (Left) Functional MAT defends against inflammation and promotes immune tolerance. The intestinal epithelial barrier is intact, commensal microbes are contained by an overlying mucus layer, and lymph vessels efficiently transport chylomicrons and lymphocytes. MAT contains healthy adipocytes and preadipocytes alongside resident immune populations, including macrophages, T cells, B cells, iNKT cells, and ILC2s. FALCs are enriched in B-1 and ILC2 cells, and MLNs harbor T cells, B cells, and ILC1–3 populations. (Right) In obesity and IBD, MAT becomes dysfunctional. An imbalance in the gut microbiome and a reduced mucus layer, caused by a poor diet or IBD, leads to increased intestinal barrier permeability and bacterial translocation. MLNs enlarge as innate and adaptive immune cells accumulate. Lymph vessels begin to branch and leak lymph, carrying antigens and other factors that trigger inflammation. Adipocytes enlarge and become insulin-resistant and inflammatory, and CLS forms around dying adipocytes. Preadipocytes acquire a profibrotic phenotype. Immune cell populations shift toward proinflammatory states, and in MAT, M2-like anti-inflammatory yet profibrotic macrophages accumulate. FALCs are infiltrated by macrophages and iNKT cells, which promote further inflammation. Creeping fat wraps around lesions in Crohn’s disease and is highly fibrotic, which contributes to intestinal strictures. Figure created using biorender.com. CLS, crown-like structures; FALC, Fat-associated lymphoid clusters; IBD, inflammatory bowel disease; ILC2s, group 2 innate lymphoid cells; iNKT, invariant natural killer T; MAT, mesenteric adipose tissue; MLN, mesenteric lymph nodes.

MAT responds dynamically to signals from its local environment, including cues from the vasculature, adipocytes, adipocyte progenitors, and resident immune populations. Obesity, impaired intestinal barrier integrity, gut dysbiosis, or IBD alter these signals, driving distinct remodeling programs within the MAT (Figure 2). Unlike the remodeling that is beneficial in acute injury, helping to contain microbial translocation and supporting epithelial repair, chronic inflammation drives maladaptive remodeling.

### 4.1 Intestinal epithelial barrier failure

Gut-associated lymphoid tissue and lamina propria immune cells provide frontline defense at the intestinal epithelial barrier, while MAT serves as a secondary firewall positioned to intercept threats that breach this barrier. The single-cell-thick intestinal epithelium faces the challenge of permitting nutrient absorption while excluding the diverse array of immunomodulatory molecules produced by the gut microbiota. When bacterial translocation or other insults occur, multiple protective mechanisms at both the barrier and within MAT prevent the development of systemic inflammation, sustain epithelial barrier function, and prevent spillover into the portal circulation. In this capacity, MAT functions as a steady, integrative organ that supports intestinal physiology rather than merely reacting to inflammatory insults.

Among adipose tissues, MAT harbors the highest quantity and diversity of bacteria. Interactions between the gut microbiota and MAT are essential for intestinal and systemic health. A healthy microbiome supports intestinal barrier integrity through several mechanisms, including the production of antimicrobial substances, the strengthening of the intestinal epithelial barrier through the production of metabolites such as short-chain fatty acids (SCFA), and the stimulation of the host’s immune system to produce protective molecules like IgA ^[22]^. These actions prevent harmful microbes from reaching the gut wall, thereby limiting their ability to invade, increasing vascularization and blood flow within the mucosa, facilitating nutrient absorption, and promoting tissue repair ^[81,82]^. However, given the MAT’s proximity to the gut and its capacity for immune surveillance, perturbations in microbial composition, termed dysbiosis, can profoundly alter the MAT’s inflammatory state and metabolic function ^[10,60]^.

It is not always clear if gut dysbiosis is a cause or a consequence of diseases such as obesity and IBD ^[83]^. Studies demonstrating that diet–microbe interactions induce small intestinal inflammation before weight gain highlight how intestinal dysfunction can precede and drive adipose pathology ^[84]^. Regardless of the cause, the presence of IBD or a diet high in fat increases fluctuations in the composition and diversity of gut bacteria, which comprise the majority of the gut microbiome ^[36]^. An imbalance in intestinal flora leads to an increase in intestinal permeability, triggering a series of inflammatory responses. Poor diet also promotes microbiota dysbiosis, which has been shown to increase gut lymphocyte trafficking into the periphery, thereby increasing the risk of atherosclerosis ^[6,20]^.

LPS, a component of the cell walls of gram-negative bacteria, is a key mediator linking dysbiosis to adipose tissue inflammation. Under conditions of increased intestinal permeability, gut-derived LPS crosses the epithelial barrier, a process facilitated in part by chylomicron binding during lipid absorption ^[85]^. Circulating LPS engages toll-like receptor 4 (TLR4) on adipocytes and immune cells, thereby activating nuclear factor kappa-light-chain-enhancer of activated B cells (NF-κB) signaling and driving the expression of proinflammatory cytokines. MAT responds to this microbial signal by activating the TLR4-regulated innate immune response. In Crohn’s disease (CD), mesenteric fat has been shown to expand in response to bacterial translocation, indicating that MAT functions as both a sensor and amplifier of gut-derived inflammatory signals ^[86]^.

### 4.2 Maladaptive expansion of MAT during obesity

During overnutrition, adipose tissue undergoes coordinated expansion and remodeling driven by interactions among adipocytes, immune cells, endothelial cells, and stromal populations ^[87]^. White adipose tissue expansion occurs through two primary mechanisms: an increase in adipocyte number (hyperplasia) and an increase in adipocyte size (hypertrophy) ^[87]^. Sustained hypertrophy is associated with local hypoxia and inflammation, driven by the production of cytokines, adipokines, angiogenic factors, and extracellular matrix components, ultimately contributing to adipose tissue dysfunction ^[87,88]^. Whether the same cues stimulate MAT expansion during obesity is unknown, particularly given MAT’s chronic exposure to gut-derived inflammatory signals that distinguish it from other depots.

From an anatomical perspective, obesogenic diets disrupt normal mesenteric vascular and lymphatic function. High-fat feeding alters blood flow regulation in the superior mesenteric artery ^[89]^ and reduces mitochondrial gene expression in mesenteric adipose endothelial cells ^[90]^. Dietary lipids accumulate within macrophages and stromal cells, reshaping the microarchitecture of MLNs ^[20,31,91,92]^. In obese mice and humans, the mesenteric lymphatic vessels become highly branched, allowing lymph to leak into the surrounding MAT, impairing the trafficking of immune cells carrying antigens from the intestine to the MLNs ^[93]^. Consistent with this, visceral lymph nodes, but not subcutaneous lymph nodes, expand in size during obesity. This is accompanied by a reduction in immune cell diversity, diminished proliferative capacity, and increased fibrosis ^[91,94]^. These physiological changes associated with obesity may lower immune resilience and increase susceptibility to the progression of IBD.

### 4.3 Inflammation in MAT is triggered by endocrine and paracrine factors

Adipose tissue in obese individuals exhibits profound alterations in adipokine and cytokine secretion, with widespread effects on multiple organ systems ^[24]^. Within MAT, several adipokines and chemokines are elevated during obesity and inflammatory stress, a response that may initially reflect attempts to counter cellular dysfunction but ultimately contributes to disease progression (Table 2) ^[15,95,109]^. Among these mediators, proinflammatory cytokines, such as TNF-α, are strongly linked to insulin resistance, type 2 diabetes, metabolic syndrome, and IBD through persistent inflammatory signaling and disruption of insulin sensitivity ^[45,99,110,111]^. Under physiological conditions, IL-6 contributes to intestinal homeostasis and immune regulation ^[112]^. However, its sustained elevation during obesity promotes increased intestinal permeability, supports T_H_17 cell differentiation, and amplifies inflammatory responses in both CD and ulcerative colitis (UC) ^[107,113]^. These effects suggest that IL-6 signaling from MAT may serve as a key link between metabolic stress and intestinal immune dysregulation.

**Table 2:** Inflammatory mediators associated with mesenteric adipose tissue.

Mediator	Primary cellular source in MAT	Immune effects	Fibrotic/structural effects	Evidence base
Leptin	Adipocytes, macrophages	Promotes T-cell proliferation; favors T_H_1 polarization; enhances macrophage activation	Indirectly supports fibrogenesis via sustained inflammation	Human CD MAT and serum, Barbier et al 2003 ^[95]^; Karmiris et al 2006 ^[96]^
Adiponectin	Adipocytes	Anti-inflammatory; suppresses macrophage activation; limits cytokine production	Anti-fibrotic; loss removes restraint on ECM deposition	Reduced in human CD MAT and serum, Rodrigues et al 2012 ^[97]^; higher gene expression in creeping fat, Yamamoto et al 2005 ^[98]^
TNF-α	Macrophages, adipocytes	Central driver of inflammation; promotes immune cell recruitment	Enhances fibroblast activation and ECM production	Strong human CD evidence; Desreumaux et al 1999 ^[99]^; validated therapeutic target, Rudrapatna et al 2025 ^[100]^
IL-6	Adipocytes, macrophages, stromal cells	Promotes macrophage recruitment; supports T_H_17 responses	Drives preadipocyte-dependent macrophage migration and fibrosis	Human MAT, Fain et al 2004 ^[101]^, Fried et al 1998 ^[102]^; mouse IBD models, Koon et al 2009 ^[103]^
Chemerin	Adipocytes, stromal cells	Chemoattractant for macrophages and dendritic cells	Promotes adipocyte progenitor activation and fibrotic remodeling	Human IBD serum, Tian et al 2025 ^[104]^; In vitro studies, Dranse et al 2018 ^[105]^
PAI-1	Adipocytes, stromal cells	Sustains inflammatory signaling	Strongly profibrotic; inhibits ECM degradation	Human abdominal visceral fat, Yildiz et al 2019 ^[106]^
TGF-β signaling	Macrophages, stromal cells	Suppresses immune resolution when chronic	Master regulator of fibroblast activation and ECM deposition	Creeping fat stromal profiling, Zhang et al 2025 ^[53]^; in vitro fibrosis studies, McGeachy et al 2007 ^[107]^
Exosomal miRNAs	Creeping fat-derived stromal cells	Modulates immune–stromal communication	Activates fibroblasts and promotes intestinal fibrosis	Human creeping fat studies (eg,miR-103a-3p); Qian, et al 2023 ^[108]^

CD, crohn’s disease; ECM, extra-cellular matrix; IBD, inflammatory bowel disease; MAT, mesenteric adipose tissue; PAI-1, plasminogen activator inhibitor-1.

Leptin is a central regulator of appetite and energy balance, as well as maintaining epithelial barrier function and influencing the polarization of immune cells, including the proliferation and survival of T cells. In obesity, however, hyperleptinemia promotes insulin resistance and systemic inflammation. Whether circulating leptin levels are consistently altered during IBD remains unresolved, with studies reporting variable results depending on disease subtype, activity, and experimental model ^[114,115]^. Interestingly, serum leptin levels are reduced in several murine models of colitis and in subsets of human IBD patients, suggesting that leptin signaling may be locally dysregulated within MAT rather than uniformly reflected in the circulation ^[96,116,117]^.

Additional adipokines produced by MAT during obesity may further shape intestinal inflammation ^[106]^. Vaspin, an insulin-sensitizing adipokine enriched in visceral fat, is elevated in UC and may represent a compensatory response to metabolic stress ^[115]^. Chemerin, although less well characterized, has been implicated in adipocyte expansion, immune cell recruitment, and inflammatory signaling, with increased serum levels reported in patients with IBD ^[104,118]^. In contrast, adiponectin levels are reduced in obese MAT, diminishing its anti-inflammatory and antifibrotic effects and contributing to metabolic dysfunction ^[97]^. However, adiponectin is paradoxically elevated in creeping fat ^[98]^. Too many Howevers! How about Numerous mechanistic studies have been performed in vitro, suggesting potential local actions of adipokines and chemokines. Nevertheless, they are challenging to interpret in the context of MAT and intestinal crosstalk.

Together, these findings suggest that MAT in response to obesity or impaired barrier function does not rely on a single dominant adipokine but instead undergoes a coordinated shift toward a proinflammatory, profibrotic endocrine profile. Reduced adiponectin, coupled with altered leptin, IL-6, and TNF-α signaling, likely acts locally, in a paracrine manner, on the adjacent intestine, while also influencing systemic immune and metabolic homeostasis. Although numerous in vitro studies support direct actions of adipokines and chemokines on immune and epithelial cells, translating these findings to the context of MAT–intestinal crosstalk remains challenging.

## 5. Mesenteric adipose tissue’s role in inflammatory bowel disease

IBD affects MAT in ways that are distinct from obesity-driven adipose remodeling. In IBD, MAT expansion and activation are not driven by excess nutrient storage but instead arise in response to intestinal inflammation and compromised epithelial barrier function. Adipocytes, immune cells, fibroblasts, and vascular-associated cells within MAT collectively contribute to localized inflammation and fibrosis ^[119]^. Although definitive cause-and-effect relationships remain difficult to establish, extensive descriptive studies, particularly in CD, demonstrate that MAT undergoes profound structural and immunological remodeling during intestinal inflammation ^[120]^.

The causes of CD are a combination of environmental and genetic factors that dysregulate host-microbial interactions, initiating and perpetuating gut inflammation in CD. In CD, dysbiosis occurs alongside disruption of intestinal barrier integrity, driven by several factors. This results in excessive activation of the immune system, leading to infiltration of immune cells into the lamina propria. As inflammation persists, commensal microorganisms and microbial products translocate into the bowel wall and surrounding tissues. Innate immune cells respond to these signals by producing cytokines and chemokines that further impair barrier function, recruit additional immune cells, and amplify adaptive immune responses. This feed-forward inflammatory loop not only perpetuates intestinal injury but also exposes adjacent MAT to sustained immune and microbial stress.

MAT’s role in UC is less defined, though it still releases inflammatory mediators, potentially contributing to UC’s systemic inflammation. Notably, other inflammatory conditions involving the mesenteric fat include mesenteric panniculitis; however, the etiology of these conditions remains unknown ^[121]^. Furthermore, while obesity is increasingly prevalent in the IBD population, its contribution to disease onset and progression remains incompletely understood. One challenge in the clinical literature is the frequent reliance on body mass index as a measure of obesity, rather than indices of visceral fat distribution, such as waist-to-hip ratio, which more closely correlate with the pathology of obesity ^[17]^. This limitation complicates efforts to understand the effects of obesity vs visceral fat on intestinal inflammation. Comprehensive reviews have summarized these clinical associations ^[122]^. The following subsections emphasize the bidirectional relationship between mesenteric fat and IBD, with evidence in the presence or absence of obesity.

### 5.1 Discovery and histopathology of creeping fat

CD provides the clearest human example of maladaptive mesenteric adipose remodeling, manifested by the development of adipose tissue that encases inflamed bowel segments. First described by Dr. Burrill B. Crohn, creeping fat is characterized by hyperplastic adipocytes, dense immune infiltration, extracellular matrix deposition, and fibrosis that correlates with disease severity and intestinal strictures, and of note, creeping fat is not seen in mouse models of IBD ^[123]^. MAT has been shown to defend against bacterial translocation and potentially attenuate intestinal inflammation in IBD ^[60]^. It has been speculated that in the early stages of CD, the interaction between creeping fat and altered microbiota may serve a protective function. The creeping fat retains and may trap the bacteria, thereby preventing their dissemination to other areas and stimulating inflammation. However, protective the intention is, as the disease progresses and the volume of creeping fat increases, this adipose tissue becomes saturated with bacteria that proliferate and migrate towards lesion sites, exacerbating the condition over time ^[54]^

Histopathological analyses reveal that creeping fat infiltrates the intestinal muscularis, comprising smaller adipocytes and an abundance of infiltrated immune cells. Fibrosis is also evident in uninvolved MAT, separate from CD lesions ^[53]^. The severity of fibrosis in creeping fat correlated positively with the thickening of the muscularis propria ^[53]^. Once established, fibrotic remodeling is largely irreversible and contributes significantly to the need for surgical resection in IBD. At least four studies have applied scRNA-seq to the stromal vascular fraction or nonadipocyte components of creeping fat ^[10,52,54,124]^. Very consistently, all four show that the creeping fat contains fewer fibroblasts but presents an expansion of endothelial and immune cells, particularly B cells, antigen-presenting macrophages, and profibrotic adipocyte progenitor cells.

### 5.2 Adipocyte transformation during IBD

MAT is a significant source of inflammatory mediators during IBD. Adipocytes and stromal cells within MAT produce a broad array of cytokines and adipokines, including IL-1β, IL-6, IL-8, IL-10, TNF-α, angiotensinogen, and plasminogen activator inhibitor-1 ^[59,125]^. The degree of cytokine expression has been shown to correlate with adipocyte mass. Importantly, adipocytes and preadipocytes express functional innate immune receptors, such as toll-like receptors and nucleotide-binding oligomerization domain-like receptors (NOD)-like receptors, enabling them to directly sense and respond to bacterial products ^[126,127]^. These signals reshape the local immune landscape and alter mesenteric lymphatic structure and function, contributing to impaired lymphatic contractility and antigen trafficking ^[110]^.

The number of adipocytes increases in creeping fat, but they are significantly smaller ^[128]^. Once creeping fat forms, the tissue loses expression of adipocyte markers, such as perilipin, and gains expression of proinflammatory markers ^[129]^. Creeping fat releases more free fatty acids from lipolysis than normal MAT. Excess free fatty acids can lead to changes in mitochondrial function, membrane phospholipid levels, redox imbalance, and inflammation. Adipocytes in creeping fat are in direct contact with intestinal smooth muscle cells. It has been demonstrated that long-chain FFAs released by creeping fat induce proliferation of smooth muscle cells in the muscularis propria ^[130]^. The strongest contributor to luminal narrowing in strictures is a thickening of the human intestinal muscularis propria. These results suggest that creeping fat may be a novel contributor to stricture formation in CD.

Rather than a single dominant adipokine, creeping fat is characterized by a shift towards inflammatory and fibrogenic signaling. Elevated leptin, TNF-α, and IL-6, coupled with reduced adiponectin, create a permissive immune environment that promotes T cell activation, macrophage recruitment, and sustained inflammation ^[109]^. In parallel, profibrotic mediators, including plasminogen activator inhibitor-1 (PAI-1), chemerin, and angiotensinogen, together with stromal-derived transforming growth factor-beta (TGF-β); extra-cellular matrix (ECM) signaling, drive extracellular matrix deposition and structural remodeling. These signals act within a macrophage–stromal–adipocyte unit, reinforcing a maladaptive state that links mesenteric fat expansion to intestinal fibrosis and disease progression in CD.

### 5.3 Stromal and adipocyte progenitor contributions to fibrosis

Hypertrophy of mesenteric fat tissue is usually shown as a marker of active CD. Stromal cells, including adipocyte progenitors and mesothelial-like cells, actively participate in growth and fibrotic remodeling of MAT. Creeping fat may form from the migration of preadipocytes out of mesenteric fat and differentiation into adipocytes in response to an increased production of fibronectin by activated muscularis propria cells ^[131]^. Inflammatory cues, such as IL-6, drive subsets of mesenchymal stem cells toward adipogenic differentiation, contributing to the accumulation of the extracellular matrix in creeping fat ^[124,125]^. There is strong evidence that specific subsets of adipocyte progenitors adopt a profibrotic phenotype in an inflammatory environment, leading to increased production of extracellular matrix, contributing to the development and maintenance of fibrotic adipose tissue ^[53,132,133]^. Data also support that creeping fat adipose stem cells secrete exosomes, which contain microRNA that contributes to intestinal fibrosis by activating fibroblasts ^[108]^. Together, these data support a model in which MAT undergoes a maladaptive transition under chronic inflammation, one that is orchestrated by stromal and immune crosstalk, reinforced by vascular remodeling, and exacerbated by systemic metabolic stress.

Vascular remodeling further contributes to MAT dysfunction in IBD. MAT is highly vascularized, and alterations in endothelial and perivascular cell function have significant consequences for tissue homeostasis. Pericytes, which are abundant in MAT and normally support vascular stability, acquire fibrotic phenotypes in inflammatory and hypoxic environments, characterized by increased extracellular matrix production ^[88]^. Consistent with this, MAT from CD patients exhibits marked hypoxia relative to healthy MAT ^[134]^. Although angiogenic pathways appear upregulated in CD-associated MAT, it remains unclear whether this vascular response adequately supports tissue expansion or instead perpetuates fibrosis and inflammation. Further investigation is needed to determine how endothelial proliferation, pericyte plasticity, and hypoxia interact to drive MAT hypertrophy and dysfunction during chronic intestinal inflammation.

### 5.4 Macrophage plasticity and fibrogenic transition

Colonic inflammation is accompanied by substantial cytokine production and immune cell infiltration in adjacent adipose tissue ^[10,60]^. Macrophages sense both commensal and pathogenic bacteria that translocate into MAT through pattern-recognition receptors and are remarkably plastic. Obesity is characterized by an accumulation of classically activated proinflammatory, M1-like macrophages in many visceral adipose depots ^[135]^. IBD-associated MAT exhibits a more complex macrophage response. Translocation of specific gut microbes into MAT during barrier disruption promotes the accumulation of macrophages with anti-inflammatory yet profibrotic phenotypes ^[6,60]^.

It is proposed that the microenvironment within the creeping fat promotes the infiltration of anti-inflammatory M2 macrophages; however, reports are inconsistent ^[12,136,137]^. Ha et al, performed single-cell RNAseq analysis coupled with functional assays to determine that bacteria polarize creeping fat macrophages to a profibrotic M2a subtype ^[10]^. Specifically, they demonstrate that *Clostridium innocuum* stimulates tissue remodeling through M2-like macrophages, in an attempt to remodel the adipose tissue ^[10]^. These M2-like macrophages are typically associated with tissue repair and resolution of inflammation, but under chronic inflammatory stress, they appear to adopt fibrogenic programs producing proinflammatory cytokines, chemokines, and toxic mediators ^[138]^. This leads to the modulation of epithelial cell proliferation, angiogenesis, and fibrosis in the intestine and its MAT. The paradoxical mechanism by which MAT-resident anti-inflammatory M2-like macrophages increase and then switch to profibrotic states under chronic inflammatory stress remains unknown.

Previous studies have highlighted that numerous IBD susceptibility genes are associated with the monocyte-macrophage lineages ^[139]^. Cytokine signaling, such as IL-6, is elevated in abdominal fat and further promotes preadipocyte-dependent macrophage migration and inflammation consistent with IL-6 as a therapeutic target in CD ^[101–103]^. In addition, macrophage-associated gene arginase-2 is increased in inflamed intestinal mucosa and MAT, and MLNs from CD patients ^[140]^. In parallel, proinflammatory CCR2-dependent macrophage subsets within MAT promote bacterial dissemination, linking intestinal inflammation to systemic immune activation ^[141]^.

Together, these findings indicate that MAT-resident macrophages are not passive bystanders but active drivers of fibrosis, immune dysregulation, and disease complications, including intestinal strictures and obstruction in CD. There is a critical need to define the molecular and functional responses of MAT-resident macrophages under inflammatory stress and determine whether targeted immunomodulation is achievable.

### 5.5 T and B lymphocytes

The immune landscape of MAT is profoundly reshaped during CD, with expansion and reorganization of adaptive immune populations. T cells are enriched within MAT, where they form a distinct immunological niche that mirrors the intestinal inflammation ^[142]^. A common feature of IBD is the infiltration of intestinal tissue by inflammatory CD4^+^ T cells. The mucosal immunity profile in CD suggests that it is a T helper type 1 cell (T_H_1)-driven disease. However, other T cell responses play a role in IBD pathology ^[143]^. Inflammatory T cells regulate the function of innate cells, including epithelial cells, fibroblasts, and phagocytes, thereby stimulating a persistent hyperresponsiveness to microbial antigens and leading to tissue injury and chronic intestinal inflammation ^[143]^. Emerging evidence suggests that these pathogenic immune programs extend beyond the intestinal wall into adjacent MAT.

Notably, human studies reveal marked segment-specific differences in adaptive immune composition within creeping fat. Ileal mesenteric fat from CD patients contains dense infiltrates of T cells, including regulatory and central memory subsets, whereas colonic mesenteric fat from Crohn’s or UC patients lacks these features ^[142]^. This suggests that MAT does not passively reflect systemic inflammation, but instead adopts specific and regional immune responses to local disease activity (Table 3). While some datasets report that T and NK cells were the most abundant immune cell type in creeping fat, there were similar overall frequencies of T and NK cells between creeping fat and adjacent MAT ^[52]^. Leptin and other adipokines produced by MAT further amplify adaptive immune responses by promoting T cell proliferation and survival, reinforcing local inflammatory circuits ^[145]^.

**Table 3:** Depot- and segment-specific adaptive immune populations in creeping fat.

Cell type	Alterations with Crohn’s disease	Effects in MAT	Reference
CD4^+^ T cells (T_H_1/T_H_17)	Enriched in ileal creeping fat	Drive inflammation; activate stromal and innate cells	Kredel et al 2019 ^[142]^
Regulatory T cells	Increased in ileal vs colonic creeping fat	Immune modulation; may limit damage	Kredel et al 2019 ^[142]^
Central memory T cells	Increased in ileal vs colonic creeping fat	Sustained immune surveillance	Kredel et al 2019 ^[142]^
NK cells	No changes (understudied)	Cytotoxic and cytokine-mediated effects	Kim et al 2024 ^[11]^
B cells	Accumulate in FALCs and tertiary lymphoid structures	Antibody production; antigen presentation	Benezech et al 2015 ^[45]^; Randolph et al 2016 ^[144]^, Kim et al 2024 ^[11]^

FALCs, fat-associated lymphoid clusters.

B lymphocytes also accumulate within MAT during CD, forming FALCs and, in some cases, tertiary lymphoid structures within creeping fat ^[45]^. These B–cell–rich aggregates may contribute to local antibody production, antigen presentation, and immune cell recruitment, although their precise role in disease progression remains incompletely defined ^[45,146]^. While B cells in visceral adipose tissue have been shown to promote insulin resistance in obesity through antibody production and T cell modulation, their role in IBD-associated MAT appears more closely tied to local immune amplification and fibrosis. Notably, B lymphocytes infiltrate MAT early during inflammatory stress, suggesting they may participate in shaping the chronic immune environment rather than merely responding to established disease ^[79,147]^.

Collectively, these findings support a model in which MAT serves as an active immunological compartment during CD, integrating adaptive immune signals that both reflect and reinforce intestinal inflammation. Nevertheless, key mechanistic questions regarding immune cell recruitment, retention, and functional polarization remain unanswered.

### 5.6 Innate lymphoid cells

ILCs participate in IBD pathogenesis through interactions with the microbiota and modulation of epithelial barrier integrity ^[148]^. ILCs within MAT contribute to epithelial support and immune regulation, but their dysregulation during chronic intestinal inflammation may promote persistent immune activation. Intestinal homeostasis requires strict regulation of the quantity and activity of local ILC subpopulations ^[148]^. While ILC2s and ILC3s are important in other fat-related conditions (like metabolic health), it is the proinflammatory ILC1s that are elevated and harmful in creeping fat. Group 1 ILCs were identified in creeping fat as a risk factor for early recurrence ^[149]^. Human mesenteric omental FALCs support rapid innate-like B-cell immune responses during infection and inflammation by producing innate antibodies ^[69]^. However, it is unclear whether these structures aid in containing bacterial translocation during CD or are present in creeping fat ^[150]^.

### 5.7 MAT involvement in ulcerative colitis: similarities and distinctions

In UC, the innate immune system becomes overactive, resulting in chronic inflammation ^[151]^. UC is associated with a T_H_2-dominated immune response, while CD is primarily driven by T_H_1 and T_H_17 responses. The clear pathological connection observed between CD and obesity is not consistently seen for UC ^[152]^. However, some research suggests a relationship between UC and obesity as aggravating factors. Additionally, evidence suggests that the expansion of a preadipocyte subpopulation in colon fibroblasts associated with UC is relevant to MAT ^[153]^. UC may not involve MAT as prominently as CD, but other aspects of immunity are at play, with distinct bacterial colonization patterns in MAT and MLNs for these types of IBD ^[9]^.

## 6. Therapeutic implications: targeting mesenteric adipose tissue

Current therapeutic approaches for IBD target the intestinal microbiome, barrier function, and aspects of immune cells, including their homing, retention, and activation ^[100]^. Targeting inflammation within adipose tissue is an emerging therapeutic concept ^[154]^. Pharmacologic and lifestyle interventions that reduce visceral fat inflammation may complement established IBD treatments and improve long-term outcomes. Strategies that limit overall adiposity, reduce MAT expansion, or dampen inflammatory signaling within this depot are likely to be beneficial. For example, newer incretin-based glucose/weight loss therapies improve adipose tissue health by reducing fat mass and inflammation. Consistent with this, weight loss following bariatric surgery has been associated with improved IBD outcomes, suggesting that modulation of visceral fat may contribute to disease amelioration ^[155]^. Although these approaches are promising, most clinical trials do not directly assess MAT, potentially overlooking a key immunometabolic mediator of therapeutic benefit. Further studies specifically evaluating how these interventions remodel MAT will be essential for understanding their full impact and for developing targeted strategies to mitigate pathological MAT inflammation in IBD.

### 6.1 Dietary fiber

Just as a poor diet increases the risk of disease, certain aspects of a healthy diet may help alleviate inflammation associated with MAT and IBD. Fiber, particularly soluble fiber, helps reduce mesenteric fat through several mechanisms, including appetite regulation, modulation of gut microbiota, and influencing fat and glucose metabolism. SCFAs are major metabolites of the intestinal flora, and they maintain intestinal barrier function by multiple mechanisms ^[156]^. Fecal SCFA levels have been reported to be lower in IBD ^[157,158]^, and low-fiber diets may put patients at risk for IBD ^[159]^. Consumption of dietary fiber has been shown to increase *Bifidobacterium* and *Lactobacillus* species, increasing production of SCFA such as acetate, propionate, and butyrate, which have anti-inflammatory properties ^[158]^. A meta-analysis by Milajerdi et al ^[160]^ found no significant association between dietary fiber intake and the risk of UC, but a significant inverse association was found between dietary fiber intake and the risk of CD. However, fruit and vegetables are inversely associated with risk of IBD and its subtypes, including UC ^[160]^. Dietary fiber improves IBD symptoms, balances inflammation, and enhances health-related quality of life ^[161]^. Nevertheless, little is understood about how fiber or other probiotics affect the microbiome, inflammation, or MAT in IBD patients at different stages or with varying symptoms ^[162]^. Given the protective roles of gut microbiota and their metabolites, strategies to restore microbial balance and SCFA production may support the management and treatment of IBD.

### 6.2 Exercise

Exercise is an emerging therapy that alters the crosstalk between adipose tissue and muscle, thereby improving inflammatory profiles associated with visceral fat. Aerobic exercise regulates mitochondrial function and lipid metabolism specifically in the MAT of obese mice ^[163]^. This is supported by data showing aerobic training transcriptionally regulates lipid metabolism genes in white adipose tissues ^[164]^. Rodent studies show that a combination of dietary intervention and exercise improves the inflammatory profile in visceral adipose tissue of obese rats ^[165,166]^. In humans and mice, exercise improves outcomes for CD ^[167,168]^. It would be fascinating to see human studies demonstrating that improving mesenteric fat specifically through exercise positively impacts IBD outcomes.

### 6.3 Browning of MAT

White adipose tissue has the capability to acquire the energy-burning characteristics of brown adipose tissue, which exhibit lipid-depleting activity and anti-inflammatory signaling profiles. Interestingly, CD is associated with browning of MAT in rodents and humans ^[169,170]^. In vitro studies using human MAT and primary mesenteric adipocytes from patients with CD and controls have demonstrated that they undergo browning. Zuo et al ^[171]^ documented that browning activation partially reduces IL-4-mediated inflammation in mesenteric responses of 2,4,6-trinitrobenzenesulfonic acid (TNBS)-treated mice. Additionally, the browning of MAT may increase secretory activity of IL-10 and IL-1 receptor antagonists, resulting in anti-inflammatory effects in colitis ^[169]^.

### 6.4 Pharmacological interventions

Drug management of IBD relies on stepwise therapies aimed at induction and maintenance of remission. Over the past two decades, biologic and small-molecule agents have significantly reshaped care, including anti-TNF antibodies, anti-integrin/anti-adhesion therapies, IL-12/23 pathway inhibitors, and janus kinase/signal transducer and activator of transcription (JAK/STAT) signaling pathway inhibitors. Despite these advances, many patients lose their response or experience adverse effects, and high costs limit the broad and prolonged use. Current goals extend beyond symptom control to include mucosal healing, deep remission, and restoration of barrier integrity, which are correlated with fewer complications and surgeries. Drugs that target inflammation within mesenteric adipose could complement established treatments and improve long-term outcomes. Novel therapies for IBD via MAT improvements are limited to studies in rodents. Examples include rosiglitazone, which ameliorates colitis in mice by inhibiting inflammation in the MATs ^[172]^. It is possible that amelioration of mesenteric adipocyte hypoxia may help attenuate CD with underlying MAT inflammation ^[134]^.

Colitis can be alleviated in mice by suppressing NLR Family Pyrin Domain Containing 3 inflammasome activation ^[173]^. Since macrophages initiate and maintain intestinal immunity, therapeutically manipulating macrophages may be an attractive approach for disease prevention and treatment. In addition, eliminating creeping fat expansion may be a strategy for enhancing the efficacy and reducing complications of CD ^[174]^. Incretin weight loss medications, such as glucagon-like peptide-1 receptor agonists, are recognized for improving adipose tissue quality by reducing fat mass and decreasing inflammation, resulting in significant metabolic improvements (cardiovascular disease, kidney disease, NAFLD) extending beyond diabetes and weight loss. We do not know the effect of incretin drugs on creeping fat. Research is just beginning; however, one study indicates that they help improve the course of IBD in obese patients ^[175]^.

### 6.5 Surgical considerations

Mesenteric fat’s involvement is not limited to the beginning stages of IBD. Data suggest that the tissue maintains inflammation in the later stages and contributes to disease relapses ^[16]^. In CD, the presence of creeping fat is a prognostic indicator for postoperative disease recurrence and increased rates of further surgery, suggesting that this fat surrounding the bowel contributes to the perpetuation of disease activity ^[176]^. Clinical trials have demonstrated that involvement of the mesentery in ileocolic resections can reduce the risk of surgical recurrence ^[177,178]^. While some studies show that more extensive removal was not different from standard mesenteric sparing surgery ^[179]^. MAT quality is also improved by a procedure called mesenteric visceral lipectomy using tissue liquefaction technology, which can reverse insulin resistance and induce weight loss in nonhuman primates^[180]^. MAT removal represents an innovative, progressive, and promising approach that appears to be highly feasible and safe for the treatment of metabolic syndrome and CD.

## 7. Conclusions

MAT is a complex cellular and physical barrier that integrates intestinal immunity, metabolism, and systemic inflammatory signaling. Its dense networks of blood vessels and lymphatics, along with direct portal drainage, enable the mucosal-associated tissue to guard the body against environmental threats originating from the gut. By sensing microbial products, buffering inflammatory signals, and supporting epithelial repair, MAT contributes to intestinal homeostasis in ways that distinguish it from other adipose depots.

In obesity and IBD, however, this adaptive remodeling shifts toward maladaptive immune activation, fibrosis, and endocrine dysfunction. Creeping fat in CD exemplifies this duality, acting simultaneously as a barrier that limits microbial spread and as a fibrotic, inflammatory tissue that promotes disease progression. Emerging single-cell and spatial profiling studies demonstrate that this transition is not driven by a single-cell type, but instead reflects coordinated remodeling across adipocytes, stromal progenitors, immune cells, vasculature, and lymphatics. The broader brain–mesentery–intestine–liver axis further emphasizes the systemic relevance of MAT ^[181]^. While neural inputs regulating intestinal barrier function and local immune tone are beginning to be defined, the mechanisms by which neural, vascular, and immune cues collectively determine whether MAT remains adaptive or becomes pathological remain poorly understood.

These findings challenge intestine-centric models of IBD and support a broader framework in which mesenteric dysfunction is not merely a consequence of disease, but a determinant of its trajectory. Despite rapid advances in profiling technologies, fundamental questions persist regarding how MAT-derived signals shape intestinal inflammation and clinical outcomes. In particular, the identity of adipokines that are causative rather than correlative remains unclear, although emerging screening approaches may enable earlier detection of CD ^[182]^. Moreover, the underrepresentation of females in clinical trials, despite sex-specific differences in adipose expandability and immune responses, represents a critical gap in the field ^[183,184]^.

The study of MAT is expected to reveal fundamental principles of immunometabolic regulation that have remained unrecognized for decades. Therapeutic strategies that preserve MAT’s barrier and reparative functions while preventing fibrosis may offer new avenues for treating IBD, metabolic dysfunction, and other chronic inflammatory disorders. Understanding MAT biology is no longer peripheral; it is central to redefining inflammation at the gut–systemic interface.

## Conflicts of interest

The author declares no conflicts of interest.

## Funding

The author is supported by NIH grant K01- DK131252 to C.M.G.
